# CB1 Expression Is Attenuated in Fallopian Tube and Decidua of Women with Ectopic Pregnancy

**DOI:** 10.1371/journal.pone.0003969

**Published:** 2008-12-18

**Authors:** Andrew W. Horne, John A. Phillips, Nicole Kane, Paula C. Lourenco, Sarah E. McDonald, Alistair R. W. Williams, Carlos Simon, Sudhansu K. Dey, Hilary O. D. Critchley

**Affiliations:** 1 Department of Reproductive and Developmental Sciences, University of Edinburgh, Centre for Reproductive Biology, Queen's Medical Research Institute, Edinburgh, United Kingdom; 2 Department of Pediatrics, Vanderbilt University School of Medicine, Nashville, Tennessee, United States of America; 3 Department of Pathology, University of Edinburgh, Royal Infirmary of Edinburgh, Edinburgh, United Kingdom; 4 Fundación IVI (FIVI)-Instituto Universitario IVI (IUIVI)-Universidad de Valencia, Valencia, Spain; 5 Division of Reproductive Sciences, Cincinnati Children's Research Foundation, Cincinnati, Ohio, United States of America; Health Canada, Canada

## Abstract

**Background:**

Embryo retention in the Fallopian tube (FT) is thought to lead to ectopic pregnancy (EP), a considerable cause of morbidity. In mice, genetic/pharmacological silencing of cannabinoid receptor *Cnr1*, encoding CB1, causes retention of embryos in the oviduct. The role of the endocannabinoids in tubal implantation in humans is not known.

**Methods and Findings:**

Timed FT biopsies (n = 18) were collected from women undergoing gynecological procedures for benign conditions. Endometrial biopsies and whole blood were collected from women undergoing surgery for EP (n = 11); management of miscarriage (n = 6), and termination of pregnancy (n = 8). Using RT-PCR and immunohistochemistry, CB1 mRNA and protein expression levels/patterns were examined in FT and endometrial biopsies. The distribution of two polymorphisms of *CNR1* was examined by TaqMan analysis of genomic DNA from the whole blood samples. In normal FT, CB1 mRNA was higher in luteal compared to follicular-phase (p<0.05). CB1 protein was located in smooth muscle of the wall and of endothelial vessels, and luminal epithelium of FT. In FT from women with EP, CB1 mRNA expression was low. CB1 mRNA expression was also significantly lower (p<0.05) in endometrium of women with EP compared to intrauterine pregnancies (IUP). Although of 1359G/A (rs1049353) polymorphisms of *CNR1* gene suggests differential distribution of genotypes between the small, available cohorts of women with EP and those with IUP, results were not statistically significant.

**Conclusions:**

CB1 mRNA shows temporal variation in expression in human FT, likely regulated by progesterone. CB1 mRNA is expressed in low levels in both the FT and endometrium of women with EP. We propose that aberrant endocannabinoid-signaling in human FT leads to EP. Furthermore, our finding of reduced mRNA expression along with a possible association between polymorphism genotypes of the *CNR1* gene and EP, suggests a possible genetic predisposition to EP that warrants replication in a larger sample pool.

## Introduction

Tubal ectopic pregnancy remains a common cause of morbidity and occasional mortality [1]. In the UK, between 2003 and 2005, early pregnancy bleeding was the third commonest cause of maternal death and over 60% of these cases were due to ruptured tubal ectopic pregnancies [2]. In the USA, ruptured tubal ectopic pregnancy remains the commonest cause of pregnancy-related first trimester death [3]. Unfortunately, our knowledge of the complex molecular and cellular interactions that contribute to tubal implantation is limited. Nevertheless, recent studies in mice have suggested that aberrant functioning of the endocannabinoid system in the oviduct leads to embryo retention and may be a cause of tubal pregnancy in women [4], [5].

Exposure to marijuana and its cannabinoid derivatives is reported to have many adverse effects on reproductive functions, including reduced fertilizing capacity of sperm, retarded development of the embryo, fetal loss and pregnancy failure [6]–[11]. Both the exogenous and endogenous cannabinoids (endocannabinoids) act through their G protein-coupled cannabinoid receptors (CB1 and CB2) but the exact mechanism by which their wide-ranging effects are mediated has yet to be defined [11], [12].

Nonetheless, in the mouse oviduct, it has been shown that a finely regulated endocannabinoid tone mediated by CB1 regulates normal oviductal transport of embryos [11]. Transport of the embryo is aided by a wave of oviduct smooth muscle movement controlled by the sympathetic nervous system [13]. Stimulation of β2-adrenergic receptors (β2-AR) causes smooth muscle relaxation and stimulation of α1-adrenergic receptors (α1-AR) causes smooth muscle contraction, leading to a wave of relaxation and contraction [13], [14]. Exposure of oviducts to either an α1-AR agonist, or a β2-AR antagonist, causes embryos to be retained in the oviduct. CB1 expression is co-localized with α1-AR, and β2-AR and oviductal nerve terminals in CB1−/− mice have increased release of norepinephrine (NE) [4]. Moreover, studies have shown that CB1−/+ embryos have normal pre-implantation development in CB1−/− oviducts but about 40% of the CB1−/− mothers still show pregnancy loss due to oviductal embryo retention [4], [15]. All of these observations have led to the proposal that CB1-mediated endocannabinoid signaling is functionally coupled to adrenergic signaling and the oviductal muscle is thought to be predominantly in a contraction (retention) phase in the absence of CB1. Although there is no evidence for implantation of embryos in the mouse oviduct, embryos can implant in the human Fallopian tube, and this could be a potential underlying mechanism for ectopic pregnancy.

**Figure 1:**
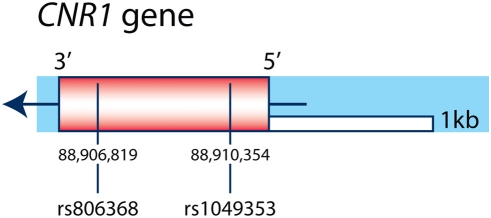
Genomic organization of *CNR1* gene and SNPs. The *CNR1* gene is a single exon gene located on chromosome 6q14–15 that is represented by a rectangle box (the orientation of the transcription of the gene is indicated by the arrow). The SNPs genotyped are represented by the vertical lines and their position in base pairs according to their chromosomal location. The names of the *CNR1* SNPs according to the NCBI database are indicated by the prefix ‘rs’.

Both adrenergic receptors have been identified in the human Fallopian tube and there is evidence of similar adrenergic control of human oviductal smooth muscle activity [16]–[18]. However, CB1 expression has not been demonstrated to our knowledge in the human Fallopian tube or endometrium. Furthermore, the suggestive differences we see in polymorphic alleles of the *CNR1* gene encoding for CB1 (see Figure 1) between cohorts of women with ectopic versus intra-uterine pregnancies needs further study to determine if they could contribute to the incidence of ectopic pregnancy [19], [20].

The aims of this study were therefore to demonstrate CB1 expression in the human Fallopian tube and endometrium from non-pregnant women and women with ectopic and intra-uterine pregnancies, and determine the distribution of *CNR1* polymorphisms in women with ectopic and intra-uterine pregnancies.

## Methods

### Tissue collection

Ethical approval for this study was obtained from Lothian Research Ethics Committee (04/S1103/20). All women were aged 18–45 years (mean 38). Written and informed consent was obtained from all patients before sample collection. Fallopian tube biopsies, endometrial biopsies (for histological dating) and sera (for measurement of circulating estradiol and progesterone concentrations for endocrine staging) were collected from women undergoing gynecological procedures for benign conditions (see Table 1) and women undergoing surgical management of tubal ectopic pregnancy. Endometrial biopsies and whole blood samples were obtained from women with viable pregnancies undergoing surgical termination of first trimester pregnancy (STOP, group 1, n = 8, mean gestation = 58.7d), from women with non-viable pregnancies undergoing surgical management of embryonic missed miscarriage in the first trimester (n = 6, group 2, mean gestation = 57.7d) and women undergoing surgical management of tubal ectopic pregnancy (n = 11, group 3, mean gestation = 58.1d). None of the women undergoing surgical management of ectopic pregnancy presented acutely with haemodynamic shock, and all required serial serum beta-HCG and ultrasound monitoring prior to diagnosis. Endometrium and trophoblast were obtained by suction curettage from groups 1 and 2. Endometrium was obtained by Pipelle™ endometrial biopsy from group 3. The decidualized endometrium was isolated from the trophoblast macroscopically in saline. Part of the Fallopian tube and endometrial biopsies were (a) immersed in RNAlater™ (Ambion, Texas, USA) at 4°C overnight then flash frozen at −70°C; (b) fixed in 10% neutral buffered formalin overnight at 4°C, stored in 70% ethanol, and wax embedded; and (c) immersed in Hanks/HEPES buffer then microwave irradiated before transfer to 10% sucrose/PBS and stored at 4 C for 30 minutes. Part (c) was then immersed in OCT freezing medium (Bayer Diagnostics) (correctly oriented in a mould), snap-frozen in liquid nitrogen and stored at −80 C before being cut into 5 um sections for immunohistochemistry (preparation of frozen specimens adapted from Slayden et al, 1995). Decidual parietalis (decidualized endometrium without trophoblast) was confirmed (using part (b) of all biopsies) by haematoxylin and eosin staining and immunolocalisation of cytokeratin prior to RT-PCR. A standard immunohistochemical protocol was used [21]. Tissue sections were incubated with mouse anti-cytokeratin primary antibody (1∶60, Dako, Ely, UK) for one hour at 37°C followed by incubation with biotinylated horse anti-mouse (1∶2000) and ABC (both Vector, Peterborough, UK) for 30 minutes at room temperature.

**Table 1:** Demographics for Fallopian tube and endometrial biopsies from women undergoing surgery for benign gynecological conditions.

Sample	Age	Parity	Cycle phase	Serum estradiol (pmol/L)	Serum progesterone (nmol/L)	Endometrium	Surgery	Reason for surgery	Uterine pathology
1	41	2	Follicular	1022.87	0.81	Proliferative	TAH	HMB	Adenomyosis
2	37	2	Follicular	940.44	3.82	Proliferative	TAH	HMB, pelvic pain	Adenomyosis
3	44	3	Follicular	829.42	4.19	Proliferative	TAH	HMB, pelvic pain	Adenomyosis, fibroid
4	36	3	Follicular	770.63	5.16	Proliferative	TAH	HMB	No histological abnormality
5	44	2	Follicular	116.3	2.88	Proliferative	STAH	HMB, dysmenorrhoea	No histological abnormality
6	34	2	Mid-luteal	471	62.65	Mid-secretory	LAVH	HMB, dysmenorrhoea	Adenomyosis, fibroid
7	40	2	Mid-luteal	242	53.1	Mid-secretory	TAH	HMB, dysmenorrhoea	No histological abnormality
8	35	2	Mid-luteal	424	76.9	Mid-secretory	TAH	pelvic pain	No histological abnormality
9	40	5	Mid-luteal	255	49.05	Mid-secretory	TAH	HMB, dysmenorrhoea	No histological abnormality
10	38	3	Mid-luteal	266	37.1	Mid-secretory	TAH	HMB	No histological abnormality
11	35	4	Menstrual	73	2.67	Menstrual	TAH	HMB, dysmenorrhoea	Adenomyosis
12	42	2	Menstrual	55	15.05	Menstrual	TAH	HMB	No histological abnormality

TAH = total abdominal hysterectomy.

STAH = sub-total hysterectomy.

LAVH = laparoscopically-assisted vaginal hysterectomy.

HMB = heavy menstrual bleeding.

### Quantitative real-time PCR

Total RNA was extracted from the Fallopian tube and endometrial biopsies as detailed in the manufacturers' protocol (Qiagen, West Sussex, UK). The concentration and quality of the extracted RNA was assessed using an Agilent bioanalyser. All samples were standardised for quality control and assigned an RNA integrity number (RIN). RNA samples were considered to be of good quality when a mean RIN value of 7.5 was obtained [22]. CB1 levels were determined by quantitative real-time PCR. This technique relates the amount of CB1 mRNA present to levels of ribosomal 18S, controlling for the amount of RNA present. Details of the RT and PCR methods have been fully detailed elsewhere [23]. Specific human CB1 primers and probe (pre-validated Taqman gene expression assay) were obtained from Applied Biosystems (Foster City, CA). Within-assay variation of PCR measurements were calculated from six replicates. The variability of the RT step was determined by reverse transcribing one RNA sample on eight separate occasions. The eight complementary DNA samples were then included within one PCR run, and variability (relative to SD) was calculated. The possibility of genomic DNA contamination was excluded by DNAase treatment and by measurement of beta-actin levels in RNA samples (which were not reverse transcribed). The relative mean of the samples from each clinical group was logged and analysed by analysis of variance (ANOVA) followed by Kruskal Wallis post hoc analysis.

### CB1 immunohistochemistry

Frozen tissue sections were fixed for 30 minutes in cold acetone at −20°C then allowed to air-dry for two hours. Sections were washed in TBS+0.5% Triton-X (TBSTX) for 10 minutes before blocking of non-specific endogenous peroxidase activity with 3% hydrogen peroxide (Sigma Aldrich, Poole, UK) in distilled water for 10 minutes at room temperature. Following a further wash in TBSTX, a non-immune blocking solution of normal goat serum was applied for 20 minutes prior to application of cannabinoid receptor 1 antibody (rabbit polyclonal, Abcam, Cambridge, UK) diluted 1∶300 in normal goat serum. Sections were incubated overnight at 4°C then washed in TBSTX. For the negative control sections, the primary antibody (diluted 1∶300) was pre-incubated in antibody diluent for three hours at room temperature with fifty times the amount of cannabinoid receptor 1 peptide (Abcam, Cambridge, UK) prior to the overnight incubation. A biotinylated goat anti-rabbit secondary antibody (Vector Laboratories, Burlingame, CA) was applied for 30 minutes at room temperature, then washed in TBSTX before a horseradish peroxidase detection system was applied for 30 minutes (ABC-Elite, Vector Laboratories). Immunoreactivity was detected using the chromagen, 3,3′-diaminobenzidine (ImmPACT DAB, Vector) followed by counterstaining of sections with Harris's haematoxylin (Pioneer Research Chemicals, Colchester, UK) and mounted in Pertex (Cell-path, Hemel Hempstead, UK).

### Extraction of genomic DNA from whole blood

Genomic DNA was purified from 2 ml frozen whole blood samples using the QIAamp Blood Midi Kit (Qiagen, West Sussex, UK) as per the manufacturers' protocol. The DNA was quantified using a ND-1000 spectrophotometer (Nanodrop technologies, DE, USA).

### PCR analysis of genomic DNA (see Figure 1)

The 1359G/A (rs1049353) single nucleotide polymorphism (SNP) encoding a synonymous Thr at amino acid 453 residue and the rs806368 C/T SNP in the 3′ UTR of the of the *CNR1* gene were genotyped using the Applied Biosystems TaqMan SNP Genotyping Assays (C_ 1652590_10 and C_8943804_10). The recommended protocols were followed using an ABI 7500 Real Time PCR System with reaction volumes of 25 rather than 50 µl and all reagent volumes reduced by 1/2. The genotypes were compared using Likelihood Ratio and Pearson analyses.

## Results

### CB1 mRNA expression in human Fallopian tube

**Figure 2:**
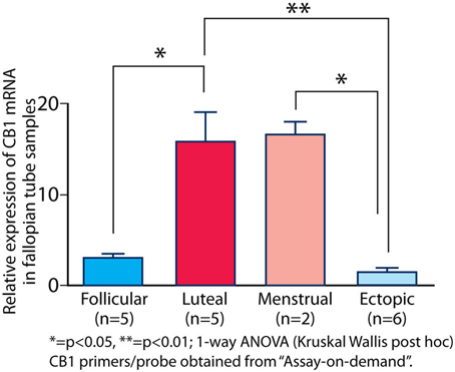
CB1 expression in the Fallopian tube. CB1 mRNA expression was lower in the follicular compared to the luteal phase. In Fallopian tube from women with ectopic pregnancy, CB1 mRNA expression was also low (p<0.05).

In the normal human Fallopian tube, CB1 mRNA was higher in the progesterone-dominant luteal phase (mean ΔC_T_ 15.42±SEM 0.42, n = 5) compared to the follicular phase (mean ΔC_T_ 18.45±SEM 0.20, n = 5) (p<0.05) (see Figure 2). In Fallopian tube from women with ectopic pregnancies (n = 6), CB1 mRNA expression was low (mean ΔC_T_ 19.17±SEM 0.41) (p<0.05), mimicking that of the Fallopian tube in the follicular phase (see Figure 2).

### CB1 mRNA expression in human decidualized endometrium

**Figure 3:**
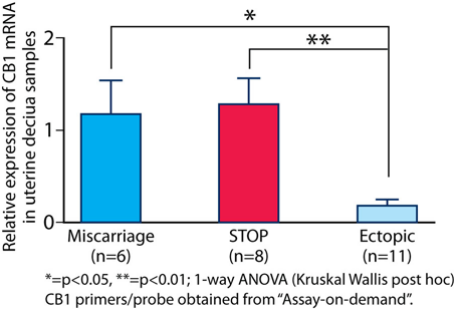
CB1 mRNA expression in the decidualized endometrium of women with ongoing pregnancies, miscarriage and ectopic pregnancies. In decidualized endometrium from women with ectopic pregnancy, CB1 mRNA expression was lower than in women with intra-uterine pregnancies (p<0.05).

CB1 mRNA expression was also significantly lower (p<0.05) in the decidualized endometrium of women with ectopic pregnancies (mean ΔC_T_ 20.32±SEM 0.47, n = 11) compared to the endometrium from intra-uterine pregnancies (viable/STOP, mean ΔC_T_ 15.84±SEM 0.22, and non-viable/miscarriage, mean ΔC_T_ 16.36±SEM 0.45, combined) (n = 14) (see Figure 3). Thus, low levels of CB1 mRNA were expressed in both the Fallopian tube and decidualized endometrium of women with tubal ectopic pregnancy.

### CB1 protein expression in human Fallopian tube and decidualized endometrium

**Figure 4:**
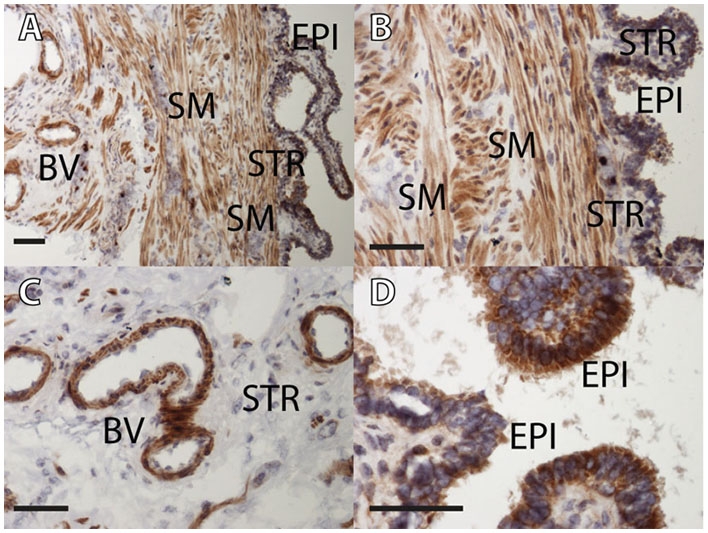
Immunohistochemistry for CB1 protein expression in Fallopian tube. CB1 protein was expressed in the smooth muscle of the wall (A and B), the smooth muscle of the endothelial vessels (A and C) and in the cytoplasm of the luminal epithelium of the Fallopian tube (A, B and D). There was no evidence of nuclear or stromal expression. EPI = epithelium, STR = stroma, SM = smooth muscle, BV = blood vessel. Scale bar = 50 microns.

CB1 protein was located in the smooth muscle of the wall, the smooth muscle of the endothelial vessels and luminal epithelium of the Fallopian tube (see Figure 4). Expression was seen in the cytoplasm of the epithelial cells but not the nuclei. There was no evidence of tubal stromal expression. CB1 protein was expressed in the epithelial cells but not the stroma of the endometrium from women with ectopic pregnancies.

### *CNR1* polymorphism distribution

**Figure 5:**
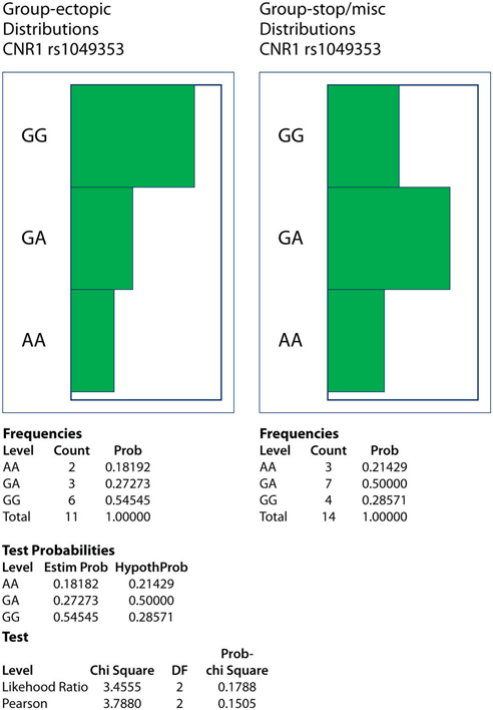
Allelic frequencies of rs1049353 (1359G/A) and rs806368 (3′UTR) single nucleotide polymorphisms (SNPs) of the *CNR1* gene in women with ectopic and intra-uterine (surgical termination of pregnancies and miscarriage groups combined) pregnancies. While rs1049353 GG homozygotes constituted 55 versus 29% of ectopic and intra-uterine cohorts, respectively, differences in genotype distributions were not statistically significant (Likelihood Ratio and Pearson analyses had p = 0.18 and 0.15, respectively). The rs806368 SNP was not informative as all ectopic and 13/14 intra-uterine subject DNAs were TT homozygotes.

The distribution of two *CNR1* SNPs (rs1049353 and rs806368) (see Figure 1) was compared in DNA samples from small cohorts of women with ectopic (n = 11) or intra-uterine pregnancies (viable and non-viable combined, n = 14). While rs1049353 GG homozygotes constituted 55 versus 29% of ectopic and intra-uterine cohorts, respectively (see Figure 5), differences in genotype distributions were not statistically significant (Likelihood Ratio and Pearson analyses yielded X^2^ of 3.5 and 3.8 (p = 0.18 and 0.15, respectively). The rs806368 SNP was not informative as all ectopic and 13/14 intra-uterine subject DNAs were TT homozygotes.

## Discussion

To date, study of the role of the endocannabinoids in female reproduction has mostly been limited to mice [10], [11]. Thus, to our knowledge, this is the first demonstration of CB1 mRNA and protein expression in human endometrium and Fallopian tube. Herein, we show differences in CB1 mRNA in the Fallopian tube and pregnant (decidualized) endometrium of women with ectopic compared to intra-uterine pregnancies providing further evidence of the importance of endocannabinoid signaling in normal implantation and other pregnancy events. Furthermore, we suggest that SNP or other variations in the gene coding for CB1, *CNR1*, may possibly contribute to a predisposition to ectopic pregnancy.

Our other finding of a temporal variation of expression of CB1 in the Fallopian tube reflects that of many genes in the endometrium suggesting a role for progesterone in the human endocannabinoid system [24], [25]. Interestingly, progesterone in the mouse uterus has been shown to regulate the production of fatty acid anandamide hydrolase, an enzyme that controls the cellular uptake and degradation of one of the major endocannabinoid ligands, anandamide (AEA) [26]. Given the detrimental effects of high anandamide levels on the embryo and the oviduct, our data further supports the idea that there is an efficient system for its tight regulation under hormonal control.

In our study, we also show that CB1 protein is expressed primarily in the smooth muscle of the wall and blood vessels of the Fallopian tube, with some expression in the cytoplasm of the luminal epithelium. This is reassuringly consistent with murine data [11]. In the mouse oviduct, CB1 mRNA and protein are expressed in the smooth muscle, conveniently co-localized with the adrenergic receptors, and it is proposed that AEA levels, mediated by CB1, regulate the release of NE to control oviduct muscle action in the mouse [4]. Our data therefore support this proposed mechanism for the control of Fallopian tube smooth muscle contractility and embryo transport in humans.

CB1 mRNA was expressed in low levels in both the Fallopian tube and pregnant endometrium of women with ectopic pregnancies. The most obvious difference in the endometrium of the women with ectopic compared to intra-uterine pregnancies is the absence of trophoblast. There are no published data to our knowledge to suggest that CB1 expression in the uterus is regulated by local trophoblast signals. Nevertheless, the coordinated down-regulation of embryonic CB1 and uterine AEA levels are important for both embryo activation and uterine receptivity (two events critical for successful implantation) so it is not inconceivable that uterine CB1 expression could be under embryonic control [15], [27]. This provides an interesting potential mechanism for the changes observed in our study. Alternatively, if the uterine environment truly reflects that of the Fallopian tube, the reduced expression of CB1 in the endometrium of women with ectopic pregnancy may simply be a consequence of extra-uterine implantation. However, it is also possible that the reduced expression of CB1 may predispose to, rather than be a consequence of, tubal pregnancy. This is supported by evidence from the studies in CB1 knockout mice but it will be difficult to design further studies to investigate this finding in humans [4].

However, our observation of suggestive differences in distribution of SNP alleles of the *CNR1* gene encoding CB1 warrants further investigation that also excludes the possibility that some subjects with ectopic pregnancy and reduced mRNA expression genotyped as 1359G/G homozygotes may actually be G/deletion heterozygotes. To date, the 1359G/A SNP is considered as the unique haplotype tagging SNP of the *CNR1* gene but a direct role of this SNP tagging of the CNR1 gene has still to be demonstrated [20]. Indeed, despite its location in a coding region, its functional role has not yet been established. Furthermore, as about twenty SNPs have been identified in the CNR1 gene, further SNPs should be tested [28]. Replication of these data in a larger sample is also needed in order to reach the conclusion that the *CNR1* gene, and indeed CB1, is important for embryo transport and successful intra-uterine implantation.

The demonstration of a potential role for CB1 in the etiology of human ectopic pregnancy is important. Cigarette smoking is a major risk factor for ectopic pregnancy and there is evidence of altered oviductal transport in rats exposed to nicotine [1], [29]. Chronic exposure of rats to nicotine affects brain endocannabinoid levels in a region-specific manner [30]. This crosstalk may also exist in the human female reproductive tract explaining the association between cigarette smoking, Fallopian tube dysfunction and ectopic pregnancy.
